# Active and sedentary behaviours in children aged 7 to 10 years old: the urban and rural contexts, Brazil

**DOI:** 10.1186/1471-2458-14-1174

**Published:** 2014-11-18

**Authors:** Flávio Andrade Neto, Fabiola Naomi Eto, Taísa Sabrina Silva Pereira, Luciana Carletti, Maria del Carmen Bisi Molina

**Affiliations:** Physical Education and Sports Centre, Federal University of Espírito Santo, Vitória, ES Brazil; Post Graduate Program of Collective Health, Federal University of Espírito Santo, Avenida Marechal Campos 1468, Maruípe, Vitória, ES 29040-090 Brazil

**Keywords:** Child, Sedentary lifestyle, Physical activity, Urban health, Rural health

## Abstract

**Background:**

Although the effects of physical activity (PA) on health and wellness are well-established, incorporating sedentary behaviours in the daily lives of populations from high- and medium-income countries is becoming increasingly common. Regardless of other factors, the area of residence can influence the physical activity level and sedentary behaviours. The aim of this study was to identify and analyse active and sedentary behaviours and factors associated with physical activity in two different geographical areas in south-eastern Brazil.

**Methods:**

1.770 schoolchildren aged 7-10 years old were studied. Parents or caregivers completed a validated questionnaire on PA and sedentary behaviours. Screen time (ST) was calculated from the time spent watching television, playing video games and using the computer. The level of active PA (>300 minutes per week) was identified and compared between the areas of study. Parametric and non-parametric tests and Poisson regression model with robust variance were used for statistical analysis.

**Results:**

Compared to schoolchildren from the urban area, those from the rural area showed a higher percentage of compliance with the PA recommendations (87 vs. 69.7%) and screen time <2 h (34.8 vs. 18.8%) and less participation in supervised PA. Active commuting to school was more common among schoolchildren from urban areas, although using a bicycle was more common in rural areas. The characteristics of children who do not meet the recommended weekly PA are as follows: being female, living in urban areas, being overweight, not using video games or performing supervised PA. Total ST as well as daily use of television and the computer was not associated with physical activity level in the present sample. Participation in supervised physical activities in both areas was found to increase the prevalence of being active in the areas studied.

**Conclusions:**

The results of the present study suggest that while schoolchildren from rural areas are more active and spend less time on sedentary activities than those from the urban area, the time spent in sedentary behaviors, such as watching television, playing video games and using the computer, is high in both contexts, and it is not associated with physical activity recommendation compliance.

## Background

Although the effects of physical activity on health and wellness are well-established, incorporating sedentary behaviours into the daily life of the population is becoming increasingly common [1], especially in children [2]. Regarding the physical activity patterns of individuals, studies have shown that the environment in which an individual lives seems to exert a direct influence on this pattern [3–5].

The relationship between physical activity and geographical context is a subject that has received less attention in middle- and low-income countries compared to studies that investigate the associations between physical activity and socioeconomic factors. In turn, environmental variables are particularly important when studying physical activity levels because the practice of physical activity depends on appropriate conditions [6].

Socioeconomic and demographic factors have a significant contribution to the variation in physical activity levels, but environmental changes may also lead to changes in the behaviour of populations and the adherence to a more or less active lifestyle [7]. Socioeconomic changes related to technological advancements also impact the lives of the population in different ways. In rural areas, this occurs through the modernisation of agriculture and animal husbandry, improved means of communication and a higher consumption of items that were previously inaccessible to these population groups [8]. Another aspect is related to urban expansion, which is associated with decreased levels of physical activity [5], a phenomenon that has been occurring at an accelerated rate in Brazil.

The physical structure of cities also reflects a paradox in behaviours related to physical activity. Certain conditions of the built environment, such as the presence of sidewalks, streetlights and the interconnectivity of pathways, seem to encourage physical activity, whereas the decreased number of open parks, increased high-speed transit routes, dependence on automotive transportation and increased social problems, such as violence, seem to discourage participation in physical activity by children [5].

Furthermore, the increase in sedentary behaviours among schoolchildren, especially in urban areas, must be emphasised. Screen time, defined as the total time spent watching television, using computers and playing video games, is a somewhat recent phenomenon but is very common among young people [2]. These behaviours, especially the time spent watching television, have also been associated with an increased risk of being overweight or obese in children and adults [9–12].

Changing certain attitudes depends on appropriate conditions and available space, especially for children, whose behaviours are usually controlled by parents or guardians [6]. A better understanding of the relationships to lifestyles expressed in rural-urban spatial interactions is necessary to propose specific policies for different contexts. Moreover, there are few studies that have been conducted in Brazilian children that have identified and analysed the differences between urban and rural areas. Thus, the present study aimed to describe and analyse active and sedentary behaviours of children aged 7 - 10 years old from different areas of residence – urban and rural – of a state in south-eastern Brazil. In addition, factors associated with physical activity will be identified in both areas.

## Methods

The present study is based on two studies on the health of children aged 7 - 10 years old living in two municipalities in south-eastern Brazil. Therefore, the present study is characterised as a cross-sectional comparative study conducted from the following databases: SAÚDES– Vitória Study and SAÚDES– Santa Maria de Jetibá Study (SMJ), both of which aimed to investigate the health, nutrition and physical activity conditions of schoolchildren aged 7 - 10 years old enrolled in public and private schools from two municipalities of the State of Espírito Santo, Brazil. Both studies were approved by the Research Ethics Committee of the Federal University of Espírito Santo (Universidade Federal do Espírito Santo, UFES), under No. 89/06 for SAÚDES–Vitória and under No. 60/09 for SAÚDES–SMJ. The participation of the children in this study was contingent on an informed consent form signed by their parents or guardians authorising their participation.

The SAÚDES–Vitória Study was conducted in the capital of Espírito Santo, one of the states in south-eastern Brazil. The total population of the state was approximately 300.000 inhabitants, and 18.000 were in the age group of the present study. The other study (SAÚDES–SMJ) was conducted in the municipality of Santa Maria de Jetibá, located in the mountainous region of the state, whose total population was approximately 30.000 inhabitants, and 1.800 were in the age group of the present study. The present study considers Vitória the “urban area” and SMJ (Santa Maria de Jetibá) the “rural area” because the economy of SMJ is based on family farming, and most of the population lives on rural properties.

The sampling procedure used was the probabilistic (stratified random) two-stage cluster sampling method. The school was the primary unit and the class was the secondary unit; the model was designed so that both municipalities were completely covered geographically according to their school zones, gender and age group of interest. In the urban municipality, the data was stratified by shares of school type (public and private), gender (male and female) and age (7, 8, 9 and 10 years old) [13]. In the rural municipality, the first stage was the selection of schools according to area (urban or rural area); in the second stage, the size of the school was considered (small: up to 50 students; medium: from 51 to 200 students; large: more than 200 students) for a proportional choice of data, according to the number of students in each school [14]. In both studies, the same protocol was used, and the data collection was performed by qualified personnel that were trained for this purpose.

Information on physical activity and sedentary behaviours was collected using a specific questionnaire for physical activity, which has been tested for its reliability [15] and validity [16]. The questionnaire consisted of questions regarding the mode and time of commuting to school, time of sedentary leisure, supervised participation in physical activities and active play, and time and daily use of the computer, video games and television. The physical activity section contained questions regarding how the child travelled from home to school (by walking or riding a bike) and the time spent walking or riding a bike during this commute. If the child travelled to school via the bus, there were questions regarding the time he/she spent from home until the bus stop and from the bus stop until school. In the same section, there was a query about the daily time spent with screen devices (TV, videogame and computer) and participating in supervised physical activity and active play. Daily screen time data was constructed from the sum of the time spent with TV, videogames and the computer.

The cut-off value for sedentary behaviour during leisure time was more than 2 hours/day, based on the WHO [17] recommended limit for entertainment-media time. The physical activity level cut-off value was also based on the WHO [17] recommendations of at least 300 minutes per week. Parents or caregivers answered a full questionnaire about socioeconomic status, health, eating habits and physical activity of the children.

Initially, the database was composed of 2.190 individuals in the age range of the study; however, 420 children were excluded because either: their parents or caregivers had not fully responded to the questionnaire for assessing physical activity and sedentary behaviour, which is an essential component of the present study, or because they had an average total physical activity time greater than or equal to 3 standard deviations from the mean. Thus, the sample comprised 1.242 schoolchildren from the SAÚDES –Vitória Study and 528 children from the SAÚDES–SMJ Study, totalling 1.770 schoolchildren. From these, 749 (44.9%) were male and 976 (55.1%) were female.

The anthropometric measurements were taken according to the recommendations of the World Health Organization [18]. Overweight and obesity were defined according to the cut-offs of the body mass index (BMI) values for specific ages and genders set by the World Health Organization [19]. The socioeconomic class was categorised according to the ABEP [20] (Brazilian Association of Research Companies) proposal, which uses the presence and quantity of household items and the education level of the household head; socioeconomic status was classified as A and B, C, and D and E.

The qualitative variables were presented as percentages using the chi-square test (X [2]) to test the hypothesis of the homogeneity of proportions and Fisher's exact test when necessary. The quantitative variables were presented as the mean and standard deviation, and the student’s t-test or the Mann-Whitney test were performed for the nonparametric distributions to examine possible differences in the variables studied. The significance level was set at 5% and was used for any of the categories of the response variable. For the multivariate analyses, a Poisson regression with robust variance was used to estimate the prevalence ratio (IRR) and confidence interval (CI 95%). Initially, variables with a p-value <0.10 were considered for the model, and the input order of the variables was the level of significance in the bivariate analyses. All analyses were conducted using SPSS (Statistical Package for the Social Sciences) version 17.0 licensed to the Federal University of Espírito Santo (Universidade Federal do Espírito Santo - UFES).

## Results

Table 1 shows the characteristics of the sample according to the residence area of the children studied – urban or rural. There were significant differences in all variables presented. The average age of the children was 8.3 ± 1.1 and 8.6 ± 1.0 (data not shown in tables) for SMJ and Vitória, respectively. A lower prevalence of being overweight and a lower maternal education was found in the rural area. In the urban area, there were higher percentages of non-white races and females.

**Table 1:** **Distribution of sociodemographic variables and nutritional status according to area of residence (urban and rural) in Brazil**

Variable	Area of residence				P-value		
	Urban		Rural			Total	
	n	%	n	%		n	%
**Sex**					< 0.01		
Male	519	41.8	275	52.1		794	44.9
Female	723	58.2	253	47.9		976	55.1
**Age (years)**					< 0.01		
7	245	19.7	157	29.7		402	22.7
8	351	28.3	141	26.7		492	27.8
9	347	27.9	125	23.7		472	26.7
10	299	24.1	105	19.9		404	22.8
**Skin colour**					< 0.001		
White	414	34.1	393	81.5		807	47.6
Non-white	798	65.8	89	18.5		887	52.4
**Mother's education (years)**					< 0.001		
≤ 4	122	10	394	75.8		516	29.7
5 to 8	348	28.6	32	6.2		380	21.9
9 to 12	516	42.4	61	11.7		577	33.2
≤12	232	19	33	6.3		265	15.2
**Socioeconomic class**							
A + B	258	23.6	20	4.3	< 0.001	278	17.8
C	386	35.3	267	57.2		653	41.8
D + E	450	41.1	180	38.5		630	40.4
**Nutritional status**					< 0.01		
Eutrophic	950	76.5	453	87.1		1403	79.6
Overweight	292	23.5	67	12.9		359	20.4

The variables related to participation in physical activity and sedentary behaviours relative to area of residence are shown in Table 2. Active commuting (on foot or by bicycle) was more frequent among schoolchildren in the urban area, both for moving from home to school and from school to home, although the bicycle was used more often for this purpose in the rural area. A small though significant difference was found in the percentage of children who watched television daily. The children from the rural area watched television (p = 0.02) more often and used video games and computers less often than the children from the urban area (p <0.001).

**Table 2:** **Variables related to physical and sedentary activities according to area of residence (urban and rural) in Brazil**

Variables	Area of residence				P-value*	Total	
	Urban		Rural				
	n	%	n	%		n	%
**Commuting from home to school**					< 0.001		
Walking	795	64	224	42.5		1019	57.6
Bus	56	4.5	61	11.6		117	6.6
Bicycle	31	2.5	65	12.3		96	5.4
Car, motorcycle or school transportation	360	29	177	33.6		537	30.4
**Commuting from school to home**					< 0.001		
Walking	809	65.1	241	45.7		1050	59.4
Bus	56	4.5	59	11.2		115	6.5
Bicycle	24	1.9	63	12		87	4.9
Car, motorcycle or school transportation	353	28.4	164	31.1		517	29.2
**Active commuting to school**					< 0.001		
Yes	826	66.5	289	54.8		1115	63
No	416	33.5	238	45.2		654	37
**Watches TV daily**					0.02		
Yes	1.201	96.1	521	98.7		1722	97.3
No	40	3.2	7	1.3		47	2.7
**Uses video games**					< 0.001		
Yes	351	28.3	94	17.8		445	25.1
No	891	71.7	434	82.2		1.325	74.9
**Uses the computer**					< 0.001		
Yes	553	44.5	92	17.4		645	36.4
No	689	55.5	436	82.6		1125	63.6
**Supervised physical activity**					< 0.001		
Yes	355	28.6	66	12.5		421	23.8
No	885	71.4	462	87.5		1347	76.2

Note: PA = Physical activity; Chi-square test; *p ≤0.05.

In the sample studied, 422 (23.8%) children participated in some type of supervised physical activity. Among the activities reported by the children from the two areas of residence, football was the most frequently cited (n = 182), followed by ballet/dance and capoeira (data not shown in table). Participation in supervised physical activities was significantly higher among children living in the urban area (urban = 28.6%, rural = 12.5%; p <0.001).

Table 3 shows the differences between the average times spent participating in physical and sedentary activities according to the area of residence. There were significant differences in most variables, except for the average weekly time of supervised activities. The children from the rural area had a higher average daily commuting time to school on foot or by bicycle and a higher average total commuting time, whereas children from the urban area had a higher average commuting time from home to the bus stop and from the bus stop to school. The children who resided in the urban area spent more time with television, video games and the computer. Regarding other variables for physical activities, children from the rural area had a longer active play time, whereas children from the urban area spent more time participating in supervised physical activities. A higher value for the total time spent participating in physical activities was reported among children from the rural area (171 minutes daily) compared to children from the urban area (128 minutes daily).

**Table 3:** **Daily time (in minutes) of active commuting to school and sedentary and active behaviours according to area of residence (urban and rural) in Brazil**

	Area of residence						
Variables	Urban			Rural			P-value*
	n	Median	Interquartile ranges	n	Median	Interquartile ranges	
**Active commuting**							
**Home - school**							
	785	10	5-15	317	15	5-30	< 0.001
**Home - bus stop**							
	72	5	3.0-6.75	108	1	1.0-3.0	< 0.001
**Bus stop – school**							
	72	5	3.0-10.0	108	2	1.0-3.0	< 0.001
**Total time (roundtrip to school)**							
	600	10	5.0-15.0	299	10	5.0-30.0	< 0.001
**Sedentary behaviour**							
**Television**	202	180	120-240	521	180	120-184	< 0.001
**Video game**	351	60	60-120	90	30	20-60	< 0.001
**Computer**	553	60	30-120	91	30	25-60	< 0.001
**Screen time**	1216	240	180-330	521	180	120-220	< 0.001
**Active behaviour**							
**Play**	918	132	90-210	479	180	120-240	< 0.001
**Supervised physical activity**							
	356	120	120-240	66	120	90-180	0.18
**Total time of physical activity**							
**Daily**	1242	120	30-195	528	180	90-242	< 0.001
**Weekly**	1242	840	191-1360	528	1260	630-1690	< 0.001

Screen time, total time for TV, video games and computer; Interquartile ranges: 25^th^ and 75^th^ percentiles.

*Mann-Whitney test for nonparametric distributions.

Table 4 shows the distribution of demographic variables, nutritional status, and active and sedentary behaviours according to physical activity level by study area. For this analysis, the groups of children classified as active or insufficiently active were compared according to the criterion of 300 minutes per week. A total of 76.5% of the children were classified as active. Significant differences regarding the physical activity levels were found by gender, mother’s education level, the use of video games and the computer and participation in supervised physical activities. The characteristics of the children who did not meet the recommended 300 minutes of physical activity per week were: female, residents of an urban area, non-white children, overweight children who did not use video games and children who did not perform supervised physical activities. It was observed that boys participated in more physical activities than girls in both areas. The practice of supervised physical activities was associated with being active in both urban and rural areas. In urban areas, the most common level of mother’s education (p = 0.004) and the daily use of video games (p = 0.03) and the computer (p = 0.001) were associated to active children and had statistically significant differences in the levels of physical activity. A screen time greater than or equal to 2 hours a day and the daily use of television and the computer were not associated with physical activity levels in the present sample.

**Table 4:** **Distribution of demographic variables, nutritional status and active and sedentary behaviours according to physical activity level**

	Physical activity level									
	Urban area					Rural area				
Variables	<300		≥300			<300		≥300		
	n	%	n	%	P-value	n	%	n	%	P-value
**Sex**					0.014					0.052
Male	127	24.5	392	75.5		27	9.8	248	90.8	
Female	223	30.8	500	69.2		39	15.4	214	84.6	
**Age (years)**					0.794					0.207
7	68	27.8	117	72.2		20	12.7	137	87.3	
8	97	37.6	254	72.4		11	7.8	130	92.2	
9	94	27.1	253	72.9		20	16.0	105	84.0	
10	91	30.4	208	69.6		15	14.3	90	85.7	
**Skin colour**					0.488					0.978
White	111	26.8	303	73.2		49	12.5	344	87.5	
Non-white	229	28.7	569	71.3		11	12.4	78	87.6	
**Mother's education (years)**					0.004					0.615
≤ 4	40	32.8	82	67.2		45	11.4	349	88.6	
5 to 8	116	33.3	232	66.7		6	18.7	26	81.3	
9 to 12	138	26.7	378	73.3		8	13.1	53	86.9	
≥12	47	20.3	185	79.7		5	15.1	28	84.9	
**Socioeconomic class**					0.069					0.054
A + B	56	21.7	202	78.3		-	-	20	100	
C	99	25.6	287	74.3		24	9.0	243	91.0	
D + E	133	29.6	317	70.4		26	14.4	154	85.6	
**Nutritional Status**					0.483					0.067
Eutrophic	87	29.8	205	70.2		13	19.4	54	88.6	
Excess weight	263	27.7	687	72.3		53	11.5	401	88.5	
**Active commuting to school**					0.334					0.831
Yes	240	29.1	586	70.9		37	12.8	252	87.2	
No	110	26.4	306	73.6		29	12.2	209	87.8	
**Watches TV daily**					0.531					0.314
Yes	336	28.0	865	72		66	12.7	455	87.3	
No	13	32.5	27	67.5		-	-	7	100	
**Daily use of video games**					0.03					0.931
Yes	78	22.2	273	72.8		12	12.8	82	87.2	
No	272	30.5	619	69.5		54	12.4	380	87.6	
	**Physical activity level**									
	**Urban Area**					**Rural Area**				
**Variables**	**<300**		**≥300**			**<300**		**≥300**		
	**n**	**%**	**n**	**%**	**P-value**	**n**	**%**	**n**	**%**	**P-value**
**Daily use of the computer**					0.001					0.862
Yes	129	23.3	424	76.7		11	12.0	81	88.0	
No	221	32.1	468	67.9		55	12.5	381	87.5	
**Supervised Physical Activity**					<0.001					0.037
Yes	56	15.8	299	84.2		3	4.6	63	95.4	
No	293	33.1	592	66.9		63	13.6	399	86.4	
**Screen time <2 h**					0.622					0.407
Compliant	69	29.5	165	70.5		20	10.9	164	89.1	
Non-compliant	281	27.9	727	72.1		46	12.4	298	87.5	

Table 5 shows the variables that remained in the final model of the Poisson regression with a robust variance for the urban and rural areas. Participation in supervised physical activities was found to increase the prevalence of being active in the areas studied.

**Table 5:** **Poisson regression model with robust variance, according to physical activity level**

	IRR	95% CI	P-value
**Urban Area**			
Supervised physical activity	0.79	0.74 – 0.84	<0.001
**Rural Area**			
Supervised physical activity	0.89	0.86 – 0.93	<0.001

Significant differences were found between genders and the following variables: commuting time to school (round-trip), use of video games and participation in supervised physical activities. Most of the children who walked to school were female, and most of those who travelled to school by bicycle were boys. A similar pattern was found when considering the return from school. Regarding the variables “use of video games” and “participation in supervised physical activities”, the results showed that compared to girls, boys used video games more often and were more engaged in the practice of supervised physical activities. Boys had a higher average daily use of video games, daily screen time, active play and supervised activities; therefore, they had a higher total time of daily and weekly physical activity than girls (data not shown in tables).

## Discussion

The results show the relationship between physical activity level and area of residence. Children residing in the rural areas were more active, spent more time commuting to school and spent less time using screen devices. Currently, access to screen devices is also accessible in rural environments and makes electronic leisure a behaviour present in both contexts regardless of the compliance to physical activity recommendations. Although technologies such as video game consoles and computers are less frequent in the homes of children living in rural areas, the television is present in both rural and urban environments, and it is the factor contributing the most to the overall total screen time in the present analysis. The strength of this argument lies in the fact that the vast majority of children from both urban and rural areas watched television every day. The greater number of individuals from urban areas above the recommendation levels for screen time can be explained mainly by the higher use of other screen devices in those areas.

Significant differences were found regarding the use of television, video games and computers when comparing schoolchildren from urban and rural areas. The children from the rural areas used more television than the children from the urban areas. Previous studies have found similar results regarding the greater use of screen devices by urban children compared with rural children [3, 21]. Although access to such technologies is now easier for families in rural areas, cultural and socioeconomic barriers seem to reduce the use of such devices in these environments. In the present study, the families that lived in the rural area were mostly low-income families, which can also be an obstacle to obtaining electronic devices.

Additionally, in the rural areas, the children helped their parents in work activities, reducing the time available for sedentary leisure activities [22]. The city of Santa Maria de Jetibá is characterised by smallholders and family agriculture, which in theory allows for the greater inclusion of children in the daily work activities of their parents. While barriers for new technologies between rural and urban areas still exist, they have shown a tendency to become increasingly blurred. There is an adaptation in the use of new technologies based on the needs of each locality. Therefore, the ownership of technology, such as the internet, which does occur for individuals residing in rural areas, needs a cultural approach because the characteristics of these new tools require the individual to have an identification in terms of speed, convenience and interface, which occurs less often in rural areas.

Although access to certain technologies is not completely analogous to the area of residence, the number of promotional and advertising strategies for certain electronic products is more evident and massive in urban environments. The reinvention of “playing” in the XXI century has promoted new ways to have fun and spend time that differ from the high energy expenditure activities present in past generations. The images, colours and sounds irradiating from new technological devices seem to captivate children in this new form of entertainment, which contributes to developing cognitive aspects [23], but in excess, may cause health risks [1]. The reduction in activities with a greater potential for caloric expenditure seems lower in rural areas, which are less subjected to reduced residence spaces and social problems, such as the lack of security found in Brazilian urban centres. The rise of new leisure artefacts does not exclude rural environments, but these areas seem to offer other attractive options for children to combine playing with exercising.

Urban schoolchildren commute to school more frequently on foot, whereas rural schoolchildren commute more frequently by bicycle, bus and motor vehicles. Although most schoolchildren from the urban area are involved in more active methods during their commute to school, the average total daily commuting time to school was significantly higher for the rural area group. This difference can be explained by the geographical distribution of schools within each area. Individuals from urban areas often have the opportunity to study at schools in their own vicinity or neighbourhood, which facilitates commuting to school on foot. In rural communities, this is not always possible because the residences are distant from each other and from schools, but in the rural municipality studied, there was school transportation for children that lived farther from schools [24].

The present study found that children from rural areas spent more time participating in active play. To better understand this result, the existing barriers that can restrict the freedom of children living in urban areas, including spaces for playing and practicing physical activities, should be identified. The lack of security and social problems that are present more specifically in certain neighbourhoods, such as the lack of paving, street lighting and appropriate spaces for leisure and the practice of physical activities, can discourage urban families from practicing physical activities [5]. Often limited by these social difficulties and the absence of public policies, parents/guardians choose to offer other types of entertainment to children who live in urban areas, thus contributing to the increased use of screen devices, resulting in the increased time spent on sedentary behaviours, according to the findings of the present study.

It was observed that in both the urban and rural areas, participation in supervised physical activities was positively associated with an increased prevalence of physical activity among children. The increased frequency of participating in supervised physical activities among children from urban areas reflects the increased availability of these services, both public and private, in medium and large cities. Vitória, the state capital, is privileged in regard to encouraging physical activity, given that the City Council offers free schools for learning physical activities, popular gyms, scholarship programs for athletes and other programs for supporting physical activities [25]. In the private sector, there are numerous different sports schools in the city. This reality contrasts with that of the municipality of Santa Maria de Jetibá, where the bureau of sports and leisure activities also offers activity options for the community, but there is a reduced number of sports schools and centres compared to the city of Vitória.

In the present study, children from the rural area spent a greater total time participating in physical activities. Similar results were found in American studies, which also showed that children from rural areas are more active than urban children [3, 26]. However, the same studies found that there was a higher percentage of overweight children in rural environments than in large cities, which differs from what was found in our study.

According to Hume et al. [21], living in a rural area is positively associated with the fact that boys meet the recommendations for screen time and physical activity, as found in the present study. Children living in rural areas may have more opportunities for active play or active transportation, as well as a reduced access to technologies such as the internet, which contributes to those children meeting the recommendations for physical activity and sedentary leisure. However, studies have shown that physical activity behaviours and screen time variables not associated [27]. Individuals who have met the physical activity recommendations can spend much of their leisure time participating in sedentary activities. This theory suggests that physical activity and sedentary behaviours are not opposite sides of the same coin, but different coins; therefore, strategies to improve these issues must behave separately.

In our study, we found that most children who did not meet the recommendations for screen time were overweight, a situation widely evidenced in the literature [28–30]. These results can be a possible cause for the higher numbers of overweight children from urban areas. These children spend more time engaged in sedentary leisure and less time engaged in physical activities. The fact that the sample from Vitória contained higher proportions of female children could impact the results because in rural areas, the sample was balanced regarding gender, and there was a lower proportion of children who did not meet the recommendations for physical activity and screen time.

Regarding the differences found between boys and girls in their activity patterns, our study complies with another study’s results that broadly describe boys as more active than girls [31]. Physical activity is a complex behaviour that can be influenced by many factors beyond biological determinants and gender. The development of individual and collective subjectivity and historical and cultural components interlocking with economic and family characteristics may all influence the decision to participate in physical activities and maintain those habits for a period of time [32]. Therefore, a future theoretical approach on culture and the family traditions of each region can be useful in understanding these differences.

There are limitations present in this study. We used open questions for the query, which could limit the estimation of values that do not exactly represent the child in reality. In order to reduce errors generated by this limitation, the query was applied by trained interviewers. Another limitation was the impossibility to accurately determine the intensity, frequency and volume of activities, a fact that restrained comparisons with other studies. This restraint is normally found in studies that use questionnaires as an instrument for physical activity measurement.

## Conclusions

The results showed that schoolchildren from the urban area spent more time with screen devices (television, video games and the computer) and less time participating in physical activities than schoolchildren from the rural area. We emphasise that regardless of the area of residence, the children showed higher average weekly physical activity times than what is recommended by the World Health Organization. The time spent on sedentary behaviours such as television, video games and the computer was high in both contexts, but it was not associated with compliance with the physical activity recommendations.

In summary, further research on this topic is needed to better elucidate the remaining issues. The progressive decrease in physical activity levels and the concomitant increase in the adoption of sedentary behaviours in childhood and in early adolescence may be associated with other potentially harmful health behaviours. Collecting data for more conclusive answers may contribute to the support of intervention strategies, particularly through longitudinal studies, which can better explain the determinants of this behaviour variance, especially in this age range.

## References

[1] Lee I, Shiroma EJ, Lobelo F, Puska P, Blair SN, Katzmarzyk PT (2012). Effect of physical inactivity on major non-communicable diseases worldwide: an analysis of burden of disease and life expectancy. Lancet.

[2] Guthold R, Cowan M, Autenrieth CS, Kann L, Riley LM (2010). Physical activity and sedentary behavior among schoolchildren: a 34-country comparison. J Pediatr.

[3] Joens-Matre RR, Welk GJ, Calabro MA, Russell DW, Nicklay E, Hensley LD (2008). Rural-Urban differences in physical activity, physical fitness, and overweight prevalence of children. Nat Rural Health Assoc.

[4] Rodrigues LP, Bezerra P, Saraiva L (2005). Influência do meio (urbano e rural) no padrão de aptidão física de rapazes de Viana do Castelo, Portugal. Rev Portuguesa de Ciências do Desporto.

[5] Lopez RP, Hynes HP (2006). Obesity, physical activity, and the urban environment: public health. Environ Health: A Global Access Scie Source.

[6] Loucaides CA, Chedzoy SM, Bennett N (2004). Differences in physical activity levels between urban and rural school children in Cyprus. Health Educ Res.

[7] Bauman AE, Reis RS, Sallis JF, Wells JC, Loos RJ, Martin BW (2012). Lancet Physical Activity Series Working Group Correlates of physical activity: why are some people physically active and others not?. Lancet.

[8] Lindner M, Ferreira ER, Souza M (2009). A exploração das ruralidades na revalorisação do espaço rural: estímulos ao desenvolvimento do turismo na quarta colônia de imigração italiana, RS – Brasil. Encuentro de Geógrafos de América Latina 2009: Caminando en una América Latina en trasformación, Montevideo.

[9] Danner FW (2008). A national longitudinal study of the association between hours of TV viewing and the trajectory of BMI growth among US children. J Pediatr Psychol.

[10] Laurson KR, Eisenmann JC, Welk GJ, Wickel EE, Gentile DA, Walsh DA (2008). Combined influence of physical activity and screen time on recommendations on childhood overweight. J Pediatr.

[11] Morales-Ruán Mdel C, Hernández-Prado B, Gómez-Acosta LM, Shamah-Levy T, Cuevas-Nasu L (2009). Obesity, overweight, screen time and physical activity in Mexican adolescents. Salud Publica Mex.

[12] Banks E (2010). Screen-time, obesity, ageing and disability: findings from 91,266 participants in the 45 and up study. Public Health Nutr.

[13] Molina MCB, Faria CP, Montero MP, Cade NV, Mill JG (2010). Fatores de risco cardiovascular em crianças de 7 a 10 anos de área urbana, Vitória, Espírito Santo, Brasil. Cad Saúde Públ.

[14] Justo GF, Quinte GC, Carletti L, Molina MCB (2012). Nutritional extremes among school children in a municipality on Brazilian countryside. Rural Remote Health.

[15] Checon K, Fonseca VM, Faria CP, Carletti L, Molina MCB (2011). Reprodutibilidade do questionário de avaliação de atividade física para crianças aplicado no Estudo SAUDES – Vitória. Rev Bras Saúde Mater Infant.

[16] Fernandes CDR (2012). Validação do questionário de atividade física em crianças de 9 e 10 anos de idade. 78f. Dissertação (Mestrado).

[17] World Health Organization: **Global recommendations on physical activity for health**. http://www.who.int/dietphysicalactivity/factsheet_recommendations/en/26180873

[18] World Health Organization (1995). Physical Status: the use and interpretation of anthropometry – Report of a WHO Expert Committee.

[19] World Health Organization (2007). Growth reference data.

[20] Associação Brasileira de Empresas de Pesquisa (2003). Critério de classificação econômica Brasil.

[21] Hume C, Salmon J, Veitch J, O'Connell E, Crawford D, Ball K (2012). Socio-demographic characteristics of children experiencing socioeconomic disadvantage who meet physical activity and screen-time recommendations: the READI study. Prev Med.

[22] Pelegrini A, Silva DAS, Petroski EL, Glaner MF (2010). Estado nutricional e fatores associados em escolares domiciliados na área rural e urbana. Rev Nut.

[23] Oliveira R, Pessoa T (2008). Benefícios Cognitivos dos Videojogos: A percepção dos jovens adultos. Actas da Conferência ZON Zon Digital Games.

[24] Egami CY, Souza RFA, Magalhães MTQ, Costa EJSC, Alves MF, Yamashita Y (2006). Congresso de Ensino e Pesquisa em Transportes 20, 2006. Anais. Panorama das Políticas Públicas do Transporte Escolar Rural.

[25] *Prefeitura Municipal de Vitória [Vitória City Council]*. Available at: http://www.vitoria.es.gov.br

[26] Liu J, Bennett KJ, Harun N, Probst JC (2008). Urban-rural differences in overweight status and physical inactivity among US children aged 10-17 years. J Rural Health.

[27] Dumith SC, Hallal PC, Menezes AMB, Araújo CL (2010). Sedentary behavior in adolescentes: the 11-year follow-up of the 1993 Pelotas (Brazil) birth cohort study. Cad Saúde Pública.

[28] Maniccia DM, Davison KK, Marshall SJ, Manganello JA, Dennison BA (2011). A meta-analysis of interventions that target children’s screen time for reduction. Pediatrics.

[29] Schmidt ME, Haines J, O'Brien A, McDonald J, Price S, Sherry B, Taveras EM (2012). Systematic Review of effective strategies for reducing screen time among young children. Obesity (Silver Spring).

[30] Duncan MJ, Vandelanotte C, Caperchione C, Hanley C, Mummery WK (2012). Temporal trends in and relationships between screen time, physical activity, overweight and obesity. BMC Public Health.

[31] Gonçalves H, Hallal P, Amorim T, Menezes A (2007). Fatores Socioculturais e nível de atividade física no início da adolescência. Rev Panam Salud Publica.

[32] Farias Júnior JC, Nahas MV, Barros MVG, Loch MR, Oliveira ESA, De Bem MFL, Lopes AS (2009). Comportamentos de risco à saúde em adolescentes no sul do Brasil: prevalência e fatores associados. Rev Panam Salud Publica.

[CR33] The pre-publication history for this paper can be accessed here: http://www.biomedcentral.com/1471-2458/14/1174/prepub

